# GeneXpert rollout in three high-burden tuberculosis countries in Africa: A review of pulmonary tuberculosis diagnosis and outcomes from 2001 to 2019

**DOI:** 10.4102/ajlm.v11i1.1811

**Published:** 2022-08-30

**Authors:** Victor Williams, Marianne Calnan, Bassey Edem, Chukwuemeka Onwuchekwa, Chika Okoro, Christine Candari, Rhodora Cruz, Kennedy Otwombe

**Affiliations:** 1 Unit of Epidemiology and Biostatistics, School of Public Health, Faculty of Health Sciences, University of the Witwatersrand, Johannesburg, South Africa; 2 University Research Co. LLC, Manila, Philippines; 3 Vaccines and Immunity Theme, MRC Unit the Gambia, London School of Hygiene and Tropical Medicine, Fajara, Gambia; 4 Barcelona Institute of Global Health (ISGlobal), Barcelona, Spain; 5 Universal Health Coverage and Communicable and Non-Communicable Diseases Cluster, World Health Organization, Owerri, Imo State, Nigeria; 6 Perinatal HIV Research Unit, Faculty of Health Sciences, University of the Witwatersrand, Johannesburg, South Africa

**Keywords:** tuberculosis, GeneXpert, Xpert MTB/RIF, Africa, interrupted time series

## Abstract

**Background:**

The rollout of GeneXpert aimed at increasing early diagnosis of tuberculosis to improve treatment outcomes and global tuberculosis targets.

**Objective:**

This study evaluated trends in tuberculosis diagnosis and outcomes pre- and post-introduction of GeneXpert in three African countries – the Democratic Republic of the Congo (DRC), Nigeria and South Africa.

**Methods:**

Data from 2001 to 2019 were extracted from the World Health Organization’s data repository. Descriptive analysis, paired *t*-tests and interrupted time series models were used.

**Results:**

Estimated tuberculosis incidence decreased from 327/100 000 to 324/100 000 in the DRC, and from 1220/100 000 to 988/100 000 in South Africa. Incidence remained at 219/100 000 in Nigeria. The tuberculosis case notification rate did not change significantly. Increases in the new case treatment success rates were statistically significant (DRC: *p* = 0.0201; Nigeria: *p* = 0.0001; South Africa: *p* = 0.0017); decreases in mortality were also statistically significant (DRC: *p* = 0.0264; Nigeria: *p* = 0.0001; South Africa: *p* < 0.0001). Time series models showed insignificant increases in new tuberculosis cases in DRC (*n* = 1856, *p* = 0.085) and Nigeria (*n* = 785, *p* = 0.555) from 2011 to 2019; and a statistically significant decrease in South Africa (*n* = 15 269, *p* = 0.006).

**Conclusion:**

Improvements in tuberculosis treatment outcomes were achieved, but little progress has been made in new case notification due to varied implementation and scale-up of GeneXpert across the three countries. Implementation barriers need to be addressed to achieve the required tuberculosis targets.

## Introduction

In December 2010, the World Health Organization (WHO) approved the Xpert MTB/RIF assay for the early detection of pulmonary tuberculosis (PTB), particularly in people living with HIV and presumptive multidrug-resistant tuberculosis (MDR-TB) clients.^1^ Previously, sputum smear microscopy was the most used method for the diagnosis of PTB in low- and middle-income countries. Based on the type and method of microscopy, the accuracy of detection varies between 20% and 80%,^2,3,4^ and depends on the technologist’s reading, the quality of the sample, and the smear staining technique. *Mycobacterium tuberculosis* culture and drug susceptibility testing, which are the gold standard for tuberculosis diagnosis and detection of MDR-TB, are time-consuming, expensive, and requires sophisticated equipment which is not available in many facilities. Therefore, active tuberculosis disease among people living with HIV who tend to have a paucibacillary disease^2^ or active MDR-TB disease is detected later, resulting in poorer tuberculosis treatment outcomes; hence, the need for a more reliable and timely means of diagnosis.

### GeneXpert for tuberculosis diagnosis

Although the GeneXpert platform (Cepheid, Sunnyvale, California, United States) was available as early as the 1990s, it was originally only used to detect anthrax.^3^ From 2006, the platform was more widely used to diagnose tuberculosis^4,5^ and, in 2010, received an endorsement from the WHO as a first-line tuberculosis diagnostic test among people living with HIV and those presumed to have MDR-TB.^1^ The Xpert MTB/RIF assay (Cepheid, Sunnyvale, California, United States) is an automated molecular (nucleic acid amplification) test for the diagnosis of tuberculosis with comparable accuracy to culture.^4,6^ It can also detect the *M. tuberculosis* complex DNA and mutations linked to rifampicin resistance (a proxy for MDR-TB) from specimens in under two hours and reduces handling of the specimen by staff.

The capability of the Xpert MTB/RIF assay to detect smear-negative tuberculosis offers an important advantage over smear microscopy, particularly for people living with HIV and children. Its ability to detect rifampicin resistance within a short period improves the prospects of timely and appropriate treatment for MDR-TB. Culture and drug susceptibility testing are, however, mandatory to complete the drug-resistance profile and to monitor treatment. The operational characteristics of the system which combines user safety, ease of use, and the potential for use as a point-of-care tool, make it an ideal system for quick diagnosis of tuberculosis. The higher cost per sample, the need for a constant power supply, and the need for regular validation are some of the challenges associated with using the system in resource-limited settings.^7,8,9^

The improved Xpert MTB/RIF Ultra (Cepheid, Sunnyvale, California, United States), which has enhanced performance in detecting *M. tuberculosis* and resistant tuberculosis, has a higher sensitivity for smear-negative tuberculosis cases resulting from HIV and tuberculosis coinfection as well as improved rifampicin resistance results. It improves case finding among smear-negative tuberculosis patients who would otherwise have been missed and potentially have transmitted tuberculosis to other people. Between 2010 and the end of 2015, 21 549 instrument modules and about 16.2 million Xpert MTB/RIF cartridges were procured by the public sector and non-governmental organisations from 122 high burden developing countries at concessionary prices.^8^

### Country adoption of GeneXpert

The End TB strategy of 2016–2035, defined lists of countries with three types of high-burden tuberculosis infection – tuberculosis alone, MDR-TB, and tuberculosis/HIV coinfection – and identified 30 countries with a high burden of tuberculosis infection.^10^ Fourteen countries, eight of which are in sub-Saharan Africa, belong to all three lists. Xpert MTB/RIF rollout in the 14 countries with high burdens of tuberculosis, tuberculosis/HIV coinfection, and MDR-TB infection has been variable. Nonetheless, evidence of the impact of the introduction of Xpert MTB on tuberculosis diagnosis and detection of drug-resistance at the population level has been demonstrated.^11,12,13^ In Africa, the three highest burden countries are the Democratic Republic of the Congo (DRC), Nigeria and South Africa. The DRC installed their first Xpert MTB/RIF assay in 2011 but has had variable scale-up due to recurrent conflict and the Ebola epidemics. By 2016, the DRC had only 226 modules with a smear-to-Xpert ratio (defined as the market penetration of Xpert within public sector organisations) of 18 and a 6% utilisation rate.^14,15^ A 2019 study indicated there was an increase in tuberculosis case-finding and rifampicin resistance among diagnosed cases; however, treatment gaps had a significant impact on the treatment outcomes for drug-resistant tuberculosis patients.^16^ Nigeria installed the first Xpert MTB/RIF machine in 2011; by 2019, it had 1560 modules, a smear-to-Xpert ratio of 2.3, and a 27% utilisation rate.^14^ An early implementation assessment conducted by Mustapha et al. in 2015 reported minimal improvements in tuberculosis case-finding due to a multitude of challenges that affected accessibility;^17^ a follow-up assessment in 2018 found the same challenges.^18^ South Africa issued national guidance recommending Xpert as the first-line diagnostic test for tuberculosis in 2011–2012, because of the high HIV burden. The first Xpert MTB/RIF was installed in 2011 and by March 2019 there were 4228 installed modules, and the country had achieved a smear-to-Xpert ratio of 0 and a 40% utilisation rate.^19^ However, Xpert was only rolled out to the rural areas between 2013 and 2015.

Due to the variable uptake of the Xpert MTB/RIF assay since its endorsement in 2010, we set out to evaluate trends in tuberculosis incidence, case notification, and tuberculosis treatment outcomes pre- and post-introduction of GeneXpert in three high PTB-burdened African countries – the DRC, Nigeria, and South Africa. The outcomes were reported between the years 2001 and 2019.

## Methods

### Ethical considerations

This study used publicly available aggregated data from the World Health Organization’s website. Identifiable data were not used and there was no direct contact with individuals, hence ethical approval was not required for this study.

### Study design

We used a trend design strategy^20^ to describe tuberculosis incidence, case notification and treatment outcome patterns before and after the introduction of GeneXpert in three selected tuberculosis high-burden countries in Africa between 2001 and 2019. The trend design uses cross-sections at two or more points in time to assess specific changes in a population over a defined period.^20^ We identified countries with a high burden of tuberculosis, tuberculosis/HIV coinfection, and MDR-TB that introduced Xpert within two years of WHO’s 2010 endorsement and were among the top 10 global high-burden lists based on the absolute number of reported tuberculosis cases^21^. Data were extracted from the updated 2020 WHO tuberculosis burden estimates^22^, which are annual estimates from the WHO, as well as data submitted to the WHO by different countries. This included 12 tuberculosis indicators that describe annual estimates of tuberculosis incidence, diagnosed tuberculosis cases and notifications, and tuberculosis treatment outcomes. These 12 indicators were selected based on the experience of the researchers that these were adequate to describe tuberculosis incidence, case notification, and tuberculosis treatment outcomes.

### Data analysis

Data were extracted in an Excel file (Microsoft Corp., Redmond, Washington, United States) and imported into Stata 15 (Stata Statistical Software: Release 15. StataCorp LP, College Station, Texas, United States) for cleaning and analysis on 26 June 2021. The years 2001–2010 were selected to study trends before, and 2011–2019 after, rollout of GeneXpert as a routine tuberculosis diagnostic test. Different indicators that describe tuberculosis incidence, tuberculosis treatment outcome, and mortality were retained for analysis^22^.

Tuberculosis incidence data per 100 000 population, treatment outcomes and mortality rates for pre- and post-GeneXpert periods were summarised by medians and interquartile ranges. Additionally, these were presented in line graphs to illustrate trends in the two periods.

A paired *t*-test was used to compare the values of each indicator to determine if there were significant differences in the values of the different indicators between the period before and after the introduction of GeneXpert within each country.

A single group interrupted time series analysis using Newey-West standard errors assessed the impact of the introduction of GeneXpert on the number of confirmed PTB cases diagnosed from 2001 to 2019 in the three countries, using 2011 as the year the programmatic implementation of Xpert began. Missing data for diagnosed PTB cases for Nigeria in 2003 were inputted using the mean value from 2002 and 2004. Statistical significance was placed at *p* < 0.05 and output from the three models are presented as a summary table and charts. The variable ‘t’ represents the time since the start of the study, ‘x2011’ represents the intervention period, ‘x_t2011’ represents the interaction of ‘_x’ and ‘_t’, and ‘Treated’ represents the post-intervention linear trend. The ‘_cons’ represent the starting level of laboratory-confirmed PTB cases. A separate sensitivity model including tuberculosis/HIV coinfection rate was done for South Africa to ascertain if the initial model may have been confounded by HIV coinfection.

## Results

### Descriptive summary

New confirmed PTB cases, the estimated number of cases, new extrapulmonary cases, and tuberculosis notification rates were higher in the DRC and Nigeria but lower in South Africa in the period after, compared to the period before, the introduction of GeneXpert (Table 1)^22^.

**TABLE 1:** Summary statistics of various tuberculosis indicators in three African countries 2001–2010, and 2011– 2019.

Variable	Pre (2001–2010)						Post (2011–2019)					
	Democratic Republic of the Congo (*N* = 10)		Nigeria (*N* = 10)		South Africa (*N* = 10)		Democratic Republic of the Congo (*N* = 9)		Nigeria (*N* = 9)		South Africa (*N* = 9)	
	Median	IQR	Median	IQR	Median	IQR	Median	IQR	Median	IQR	Median	IQR
New pulmonary confirmed TB cases	65 040	62 192–69 477	42 383	34 402–45 721	133 352	125 864–139 136	76 671	71 526–93 555	54 012	52 811–73 660	132 750	129 840–155 473
Estimated number of cases	182 000	168 000–198 000	308 000	289 000–329 000	594 500	499 000–632 000	247 000	233 000–262 000	397 000	376 000–418 000	547 000	421 000–593 000
Estimated incidence /100k	327	327–327	219	219–219	1220	1070–1260	324	322–326	219	219–219	988	738–1110
New extrapulmonary cases	18 471	18 213–19 654	2906	1525–3422	43 193	37 686–48 251	21 579	20 329–24 959	4546	4227–4896	28 507	23 621–37 709
Lab confirmed RR/MDR	-	-	-	-	-	-	737	499–893	1981	1241–2286	17 360	13 199–19 073
Lab confirmed MDR-TB	87	87–87	21	21–21	7386	7386–7386	137	81–217	656	107–996	8981	8081–9956
TB case notification (Numb)	96 371	80 011–104 861	67 362	45 842–84 121	286 646	227 320–348 241	119 213	112 439–150 085	94 825	87 211–102 387	287 224	237 045–312 380
Case notification rate 100k	173	156–177	48	37–56	595	487–693	165	158–184	53	52–55	519	422–582
TB case detection rate (CDR) %	53	48–54	22	17–26	50	46–55	50	48–57	24	24–25	53	53–58
Estimated TB/HIV %	22	21–23	21	20–25	66	65–69	16	12–19	17	14–22	60	59–62
Estimated mortality /100k	84	83–94	88	88–93	395	371–422	87	70–89	85	81–86	116	106–137
TSR new cases %	85	78–86	79	76–81	68	65–70	89	88–90	86	86–87	78	77–80

*Source*: Adapted from World Health Organization (WHO). Global Tuberculosis Programme. In Global TB report [homepage on the Internet]. 2021 [cited 2021 Jun 26]. Available from: https://www.who.int/tb/country/data/download/en/

IQR, interquartile range; RR, rifampicin resistant; TSR, treatment success rate (defined as the proportion of cases registered in a particular year that successfully completed tuberculosis treatment without evidence of bacteriological failure [WHO]); CDR, case detection rate (defined as the proportion of estimated new and relapse tuberculosis cases detected in a given year under the internationally recommended tuberculosis control strategy [WHO]).

The estimated tuberculosis incidence per 100 000 population was lower in the DRC (324/100 000) and South Africa (988/100 000) post-introduction of GeneXpert, but remained the same in Nigeria (219/100 000). Data on laboratory-confirmed rifampicin-resistant/MDR-TB was not available before the introduction of GeneXpert. However, there was an increase in diagnosed MDR-TB cases across the three countries after the introduction of GeneXpert (137 for the DRC, 656 for Nigeria, and 8981 for South Africa). The tuberculosis case detection rate was comparable across the two periods in all three countries, while a decrease was observed in tuberculosis/HIV coinfection incidence post the introduction of GeneXpert (16% for the DRC, 17% for Nigeria, and 60% for South Africa). The estimated tuberculosis mortality showed a downward trend in all three countries with the largest decline in South Africa, from 395/100 000 to 116/100 000, after the introduction of GeneXpert. The treatment success rate in all three countries increased after the introduction of GeneXpert compared to the period before.

### Trends in select tuberculosis indicators

**FIGURE 1:**
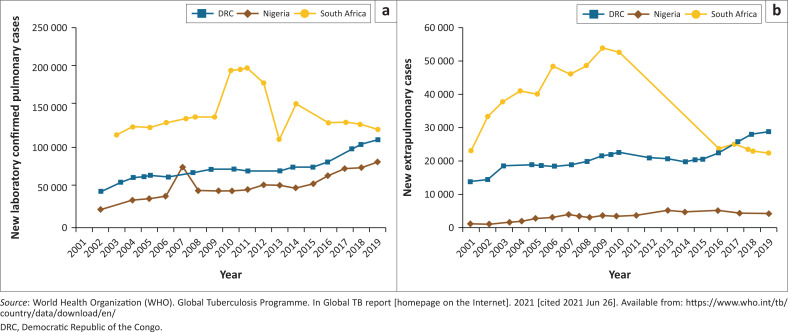
Tuberculosis cases in three countries in Africa 2001–2019 (a) New laboratory-confirmed cases 2001–2019; (b) New extrapulmonary cases 2001–2019.

Confirmed PTB cases (Figure 1a)^22^ increased gradually from less than 50 000 cases in 2002 to slightly above 100 000 in the DRC and about 80 000 in Nigeria in 2019. However, in South Africa, tuberculosis cases peaked at about 200 000 in 2011, dropping to about 120 000 in 2019. New extrapulmonary cases (Figure 1b)^22^ followed a similar pattern to confirmed pulmonary cases but with much lower numbers; Nigeria showed a slight decline in the number of cases reported between 2017 and 2019.

**FIGURE 2:**
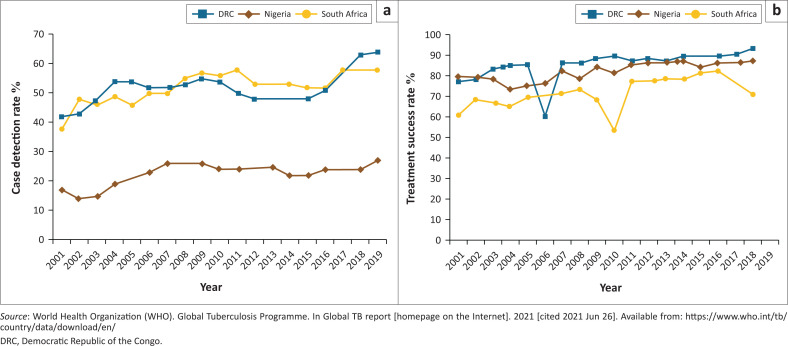
Key tuberculosis indicators in three African countries 2001–2019 (a) Case detection rate (%) 2001–2019; (b) Treatment success rate (%) 2001–2019.

The case detection rate (Figure 2a)^22^ and treatment success rate (Figure 2b)^22^ showed a slow increase from 2001 to 2019 in all three countries. Nigeria had the lowest case detection rate with the least increase. The DRC showed a sharp decrease in treatment success rate in 2006, as did South Africa in 2010, with South Africa showing another decline after 2017. There was a marked increase in tuberculosis case detection rate in the DRC and South Africa from 2016, with a leveling off by 2019.

**FIGURE 3:**
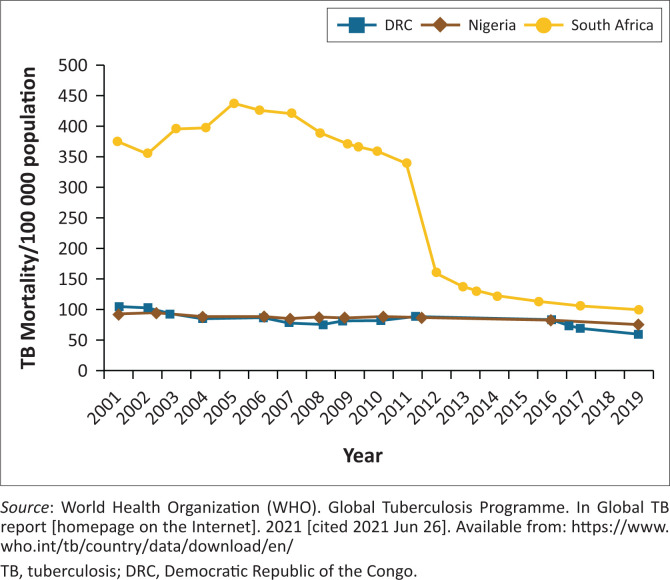
Tuberculosis-related mortality in three high-burden African countries 2001–2019.

South Africa showed the most substantial decline in tuberculosis mortality per 100 000 population (Figure 3)^22^ from 2001 to 2019, with the steepest decline occurring between 2011 and 2012.

### Comparison between the two periods

Data for rifampicin-resistant tuberculosis and MDR-TB were not available before the introduction of GeneXpert; hence no comparison was done. A statistically significant increase was seen in the new pulmonary confirmed tuberculosis cases and the estimated number of all cases in DRC (*p* = 0.0002, *p* < 0.0001) and Nigeria (*p* = 0.0011, *p* < 0.0001) but not in South Africa (*p* = 0.7413, *p* = 0.5081) (Table 2)^22^. Estimated tuberculosis incidence per 100 000 population decreased in the DRC (*p* = 0.0067) and South Africa (*p* = 0.1066), but there was no change in Nigeria. The percentage of tuberculosis/HIV incidence and mortality per 100 000 population showed a statistically significant decrease in all three countries (DRC, *p* = 0.0264; Nigeria and South Africa, *p* < 0.0001), while the percentage treatment success rate for new cases showed a statistically significant increase in the three countries (DRC, *p* = 0.0201; Nigeria, *p* < 0.0001; South Africa, *p* = 0.0017).

**TABLE 2:** Comparison of tuberculosis indicators pre- (2001–2010) and post-introduction (2011–2019) of GeneXpert in three high tuberculosis burden countries in Africa.

Variable	Democratic Republic of the Congo		Nigeria		South Africa	
	Difference	*p*	Difference	*p*	Difference	*p*
New confirmed pulmonary tuberculosis cases	23 358	0.0002	22 335	0.0011	2125	0.7413
Estimated number of cases	67 667	< 0.0001	92 222	< 0.0001	−42 889	0.5081
Estimated incidence/100k	−3.1	0.0067	-	-	−217	0.1066
New extrapulmonary cases	4994	0.0007	2134	0.0006	−9551	0.1544
Laboratory confirmed RR/MDR-TB†		-		-		-
Laboratory confirmed MDR-TB†		-		-		-
Tuberculosis case notification numbers	41 726	0.0001	32 348	0.0001	6084	0.8818
Case notification rate/100k	7.8	0.2634	7.6	0.0680	−57.1	0.4901
Tuberculosis case detection rate %	2.7	0.2305	3.3	0.0743	6.2	0.0108
Estimated tuberculosis/HIV %	−4.5	0.0190	−3.9	0.1604	−6.6	< 0.0001
Estimated mortality/100k	−8.2	0.0264	−6.0	0.0001	−254	< 0.0001
TSR new cases %	9.0	0.0201	8.4	0.0001	9.6	0.0017

*Source*: World Health Organization (WHO). Global Tuberculosis Programme. In Global TB report [homepage on the Internet]. 2021 [cited 2021 Jun 26]. Available from: https://www.who.int/tb/country/data/download/en/

MDR-TB, multidrug-resistant tuberculosis; TSR, treatment success rate; RR, rifampicin resistant tuberculosis

† , No data to enable comparison.

### Interrupted time series models

In the DRC, the initial number of laboratory-confirmed cases of PTB was 50 747, and the number of cases increased significantly every year before 2011 by 3178 (*p* < 0.0001) (Table 3, Supplementary Figures 1-3). In the first year of the intervention, cases of PTB decreased by 15 521 (*p* = 0.009), followed by a marginal increase in the yearly number of cases of diagnosed PTB (relative to the pre-intervention period) of 1856 (*p* = 0.085). After introducing the intervention, the post-trend estimate shows the cases of diagnosed PTB increased annually by 5034 (*p* ≤ 0.001). A similar trend was seen in Nigeria except for the reduction in the number of cases in the year the intervention commenced, and the increase afterwards was not statistically significant. However, the post-trend estimate showed a statistically significant increase in cases of diagnosed PTB annually by 4295 (*p* ≤ 0.001). South Africa showed a similar trend of a statistically significant annual increase of 7996 (*p* = 0.013) before 2011. There was a decline in diagnosed PTB cases by 157 in the first year of the intervention period (*p* = 0.994), however, this decrease was statistically significant annually after the intervention period by 15 269 cases (*p* = 0.006). The post-intervention linear trend also showed a significant decrease in cases of 7273 (*p* = 0.028).

**TABLE 3:** Summary of interrupted time series models output for the three highest burden tuberculosis countries in Africa 2001–2019.

New pulmonary confirmed cases	Democratic Republic of the Congo		Nigeria		South Africa	
	Coefficient	*p*	Coefficient	*p*	Coefficient	*p*
Yearly cases before 2011	3178	< 0.001	3509	0.015	7996	0.013
Cases in the first year of the intervention	−15 521	0.009	−14 649	0.188	−157	0.994
Annual cases of PTB after 2011 relative to the period prior	1856	0.085	785	0.555	−15 269	0.006
Baseline number of laboratory-confirmed PTB cases	50 747	< 0.001	27 173	< 0.001	110 712	< 0.001
Yearly cases after the introduction of GeneXpert	5034	< 0.001	4295	< 0.001	−7273	0.028

PTB, pulmonary tuberculosis

The models were not influenced by seasonality or any time-varying confounder. There was no over-dispersion as our outcome variable was continuous. An autocorrelation test was done for each of the three models up to seven lags, and there was no autocorrelation in any of the models. A separate model to ascertain the impact of HIV coinfection, which could have influenced the model output in South Africa, indicated similar findings (Online Supplementary Figure 4).

## Discussion

Our analysis showed that an increase in the number of diagnosed tuberculosis cases in the DRC and Nigeria was statistically significant, but the number of cases diagnosed in South Africa after the introduction of GeneXpert had decreased. Tuberculosis case notification rates and case detection rates did not show major changes. There was, however, a significant reduction in tuberculosis/HIV coinfection and mortality per 100 000 population, with an increase in treatment success rate for new cases in all three countries.

While all three countries installed their first Xpert MTB/RIF in 2011, there was variable adoption of Xpert as a first-line diagnostic test, inconsistent scale-up and inadequate utilisation of Xpert testing in each country.^11,15^ These factors may have considerably affected the case notification achievements of the three countries as shown in our study. In the DRC, initial evaluation post rollout indicated GeneXpert improved detection of tuberculosis cases and rifampicin resistance in patients with smear-negative sputum.^9^ However, later evaluation reports indicated the coverage was low, with one Xpert instrument serving 3.3 zones in the country.^16^ This was further complicated by recurrent stockouts and expiry of instrument cartridges as of 2018, although measures had been instituted to address these with additional staff complements and training.^23^

This study showed case notification and detection rates did not change considerably in Nigeria, with no difference in incidence rate between the two periods. This was corroborated by a 2019 assessment of GeneXpert performance, which reported low case findings (about 25% of estimated cases) despite the increase in Xpert modules and use of Xpert as the main diagnostic tool in 2016 (Xpert4all initiative).^18^ This may be because many health workers do not follow the standard protocol when a patient presents with a cough, without considering tuberculosis or ordering GeneXpert testing. In addition, there is under-utilisation of GeneXpert because of modular failures, poor power supply, inefficient sample transport mechanisms, weak data management and inadequate human resources to staff the remote test sites. However, the national programme did report an increase in tuberculosis case finding in those areas with access to Xpert.^18^ Additional studies on the utility of Xpert in Nigeria found that it increased tuberculosis case detection among smear-negative PTB patients.^24^

Implementation data from the South African National Health Laboratory services from 2011 to 2019 demonstrateed a declining trend in the number of cases detected.^19^ This is consistent with what we have reported. In a study to assess the impact of Xpert rollout on tuberculosis diagnostic yield and time to initiate therapy, the researchers observed marginal increases in tuberculosis case detection and time to initiate treatment, suggesting the need to address the health system to improve Xpert MTB/RIF efficiency.^25^ In a similar study, Xpert increased the proportion of rifampicin-resistant confirmed tuberculosis and significantly reduced the time to diagnosis, commencing therapy, and the pre-treatment mortality in patients with drug-resistant tuberculosis.^26^ However, on-treatment mortality and treatment outcomes did not change, reflecting a need to address treatment gaps and treatment outcomes.^26^

Similar to our findings, the Health Systems Trust (an organisation established to assist with the transformation of the healthcare system in South Africa) showed there has been an annual decline in tuberculosis incidence in South Africa from 2011 to 2015 and data from the University of KwaZulu-Natal suggest that the rapid drop in tuberculosis cases detected could be attributed partially to the scale-up of antiretroviral therapy that took place between 2009 and 2014.^27,28,29^ However, about 150 000 tuberculosis cases are missed annually in South Africa – nearly 50% of the tuberculosis burden in the country.^30^ Patient-side and health system-side factors may contribute to the missed cases and thus reduce the impact of Xpert on tuberculosis case detection and treatment outcomes. Major health system barriers include laboratory-running challenges such as intermittent electricity supply and limited number of Xpert cartridges, insufficient human resources, poor provider knowledge and skills to adhere to diagnostic guidelines and inadequate information management.^30,31,32^ On the patient side, barriers include loss to follow-up due to delayed diagnosis or family and work commitments, negative public sector care perceptions, and using private-sector care with limited financial resources.^33,34^

In modelling studies to determine the long-term outcome of scaling up the Xpert platform with an algorithm that used Xpert as the first-line diagnostic test, it was determined that if implemented optimally, the number of tuberculosis cases diagnosed per year would increase by 30% – 37%, while diagnosed MDR-TB cases would increase by 69% – 71%. Eighty-one percent of the cases would be diagnosed after the initial visit compared to 46% at the time.^35^ The data from each of the three countries, while showing a modest rise in the number of tuberculosis cases diagnosed, did not meet the expectations of the modelling studies. Despite the observed increases in the DRC and Nigeria, there are numerous health system challenges to the optimal rollout and utilisation of GeneXpert in the diagnosis of tuberculosis cases. South Africa registered a decline in the number of tuberculosis cases diagnosed. Variable scale-up, low utilisation rates and unaddressed patient-side and health-system barriers have negatively impacted the expected increase in tuberculosis case detection and rifampicin resistance detection, as well as treatment outcomes. Xpert performance in other tuberculosis high-burden countries reflects similar trends for case detection and treatment success.^15,36^

### Limitations

This study was not without limitations. Firstly, we used data extracted from the WHO website for which we had no control in terms of how data was collected. This study did not have a mechanism for identifying data reported to the WHO that was not updated. There were missing data for some outcomes and variables but where feasible, missing data were imputed. However, the mean imputation approach used may not have yielded the optimal value, although it provided an estimate to work with. The output of our interrupted time series analysis may have been influenced by unmeasured confounders, particularly in South Africa where the rollout of the Antiretroviral Therapy Program may have impacted diagnosed tuberculosis cases. To address this, we conducted a sensitivity analysis to ascertain if HIV coinfection impacted the South African model. Noting that interrupted time series models are generally not affected by typical confounders such as age distribution or socioeconomic status, like in the modelling of individual patient data, we believe the modelling approach was appropriate.^37^ Nevertheless, this study has highlighted the key successes since the introduction of GeneXpert for tuberculosis diagnosis and the areas requiring improvement to be on track to end tuberculosis by 2035.

### Conclusion

While the GeneXpert testing platform has been lauded as a revolutionary tool to enhance tuberculosis case finding in meeting the End TB targets,^10^ tuberculosis case notification remains low in the three highest tuberculosis burden countries in Africa. If low- and middle-income countries’ health systems do not address implementation challenges related to access and utilisation, the advantages of the Xpert diagnostic platform will not be realised and these targets will be missed.
